# The Impact of the Sanctions Made Against Iran on Availability to Asthma Medicines in Tehran

**Published:** 2016

**Authors:** Golbarg Ghiasi, Arash Rashidian, Abbas Kebriaeezadeh, Jamshid Salamzadeh

**Affiliations:** a *Department of Pharmacoeconomics and Pharmaceutical Administration, Faculty of Pharmacy, Tehran University of Medical Sciences, Tehran, Iran. *; b *Department of Health Management and Economics, School of Public Health, Tehran University of Medical Sciences. *; c *Department of Pharmacoeconomics and Pharmaceutical Administration, Faculty of Pharmacy, Tehran University of Medical Sciences, Tehran, Iran. Department of Toxicology and Pharmacology, Faculty of Pharmacy and Pharmaceutical Sciences Research Center, Tehran University of Medical Sciences, Tehran, Iran*; d *Department of Pharmacoeconomics and Pharmaceutical Management, School of Pharmacy, Shahid Beheshti University of Medical Sciences, Tehran, Iran.*

**Keywords:** Sanction, Availability, Asthma Medicines, Pharmacies

## Abstract

The impact of the international sanctions on the Central Bank of Iran in 2013 and also accessibility of medicines in this country have received a lot of media coverage. In this study we used the data collected from a group of pharmacies all located in Tehran to assess the potential effects of the banking sanctions on access to asthma medicines. Data were collected from forty community pharmacies in Tehran, using a standard methodology proposed by the WHO and Health Action International. Data were collected in two stages: first before the sanctions were made against the banking system in the summer of 2012, and second after they were in effect in the summer of 2013, and they were analyzed using univariate analysis techniques. Several imported medicines were already in shortage during 2012. As a result of the sanctions, the availability of both imported and locally manufactured asthma medicines decreased by 19% and 42%, respectively. While before the height of the sanctions 60% of the pharmacies could provide all the essential asthma medicines, this number reduced to 28% after the sanctions (p-value: 0.003). While studies about “access to medicines” in Iran prior to 2011 were indicating appropriate access, our findings suggested that the availability of asthma medicines in community pharmacies was already less than ideal in 2012 and declined dramatically after the latest wave of the sanctions. Our findings show the important effects of the sanctions on availability of asthma medications in community pharmacies.

## Introduction

 One of the Millennium Development Goals (MGD) worldwide and also in the nation for 2015 is the provision of access to essential medicines (1) . Given the current agenda of ‹universal health coverage› as the next goal of MDG, improving access to medicines must be considered a global responsibility. Common access to medicines encompasses different elements including: availability of medicines in public and private facilities, affordability of medicines for the users achieved via appropriate pricing, rational use of medicines through an effective health care system, and a sustainable financial system that ensures continuity of access to medicines (2, 3). 

Different studies have shown that health care systems have varying levels of difficulty in ensuring the elements of access; that is to say many low and middle-income countries suffer from disorganized health care systems that makes it difficult for them to ensure access to required medicines. There are examples of middle-income countries that have benefitted from a relatively stable health care system, despite their limitations (4). Studies in Iran, as an example, have demonstrated an acceptable level of availability of affordable essential medicines (5). Iran had achieved this through an established network of public and private health care providers, and a national system of medicine registration and pricing, and a supporting environment for local production of generic drugs in the country.

Ensuring access to medicines relies on the underlying general economic status of a country. Many countries in Europe reported hardship in access to medicines as a result of the economic turmoil that affected Europe from 2002 to 2011 (6). In the past decade, Iran has been the target of several international economic sanctions. These sanctions were augmented with the direct sanction of the Central Bank of Iran. The latter means that the country had a hard time receiving the money earned from exporting goods, and also paying to import the goods required in the country, that is, if the goods were not targeted by the sanctions. It has been argued in various studies that in such situations the countries find that the currency used for importing the essential goods is not accessible, as the bank accounts are blocked. While several publications from Iran and other parts of the world suggested that the medicine market had been negatively affected by the sanctions, most of these reports were based on individual data or assessments of certain medicines (7).

Asthma is a chronic disease and a global public health problem afflicting more than 300 million people throughout the world (8). Asthma incurs considerable costs to the public due to the increased mortality rate (9). However, asthma can be effectively managed, and appropriate use of medicines is an essential element in managing it. The goal of treatment is to control the disease, which is composed of two components: limiting the current impairment and reducing the risk for future deterioration and exacerbation (10).

Some studies on the impact of sanctions in similar countries have mentioned a significant increase in suffering and death due to insufficient access to essential medicines and medical equipment as consequences of those sanctions. Despite humanitarian goods such as food and drugs are excluded from the sanctions, they are not immune from the aftermath of this issue (11). Thus, considering the role of asthma medicines in reducing the symptoms of this disease and consequently increasing the quality of life in the patients, the current work investigates the effects of the sanctions made against Iran in terms of availability to asthma medicines before and after them. 

In this study we used an opportunity to assess availability of asthma medicines in several pharmacies of Tehran before and after the height of the economic sanctions in the country. 

**Table 1:** Availability of imported asthma medicines at community pharmacies in Tehran, before and after the economic sanctions

**P value for absolute difference**	**Relative difference**	**Absolute difference**	**March 2013**	**July 2012**	**Medicines**
0.06	85.7%	15%	2/5%	17/5%	Cromolyn spray 2% (Cromolex)
002	66.6%	35%	17.5%	52.5%	Beclomethasone (any product)*
<0.001	68%	42.5%	20%	62.5%	Inhaled corticosteroids (any medicine)**
0.048	77.7%	17.5%	5%	22.5%	Leukoterians (any medicine) $

* Includes Beclam, Beclex and Clenil brand medicines;

** Include Beclomethasone and Fluticasone brand medicines: Beclam, Beclex, Clenil, Flutizox and seretide; $Includes Singular and Acolate brand medicines.

**Table 2:** Availability of locally manufactured asthma medicines at community pharmacies in Tehran before and after the sanctions

**P value** **For absolute difference**	**Relative difference**	**Absolute difference**	**March 2013**	**July 2012**	**Medicines**
0.65	17.6	7.5 %	35%	42.5%	Cromolyn spray 2%
0.02	45.4%	25%	30%	55%	Inhaled Salbutamol
0.004	48.1%	32.5	35%	67.5%	Inhaled Beta-agonists
0.056	47.05%	20%	22.5%	42.5%	Inhaled Generic Corticosteroids
0.002	61.9%	32.5%	20%	52.5%	All Inhaled Antimuscarinic
0.056	20 %	20%	22.5%	42.5%	Generic Interlukins

**Table 3:** Overall availability of asthma medicine at local pharmacies in Tehran before and after the sanctions

**P value** **For absolute difference**	**Relative difference**	**Absolute difference**	**March 2013**	**July 2012**	**Medicines**
<0.0001	59.3 %	47.5%	32.5%	80%	All Inhaled Corticosteroids
0.003	54.1%	32.5%	27.5%	60%	Essential Asthma Medicines

## Experimental

*Material and Methods*

 This was a before-after study, involving field data collection from 40 community pharmacies located in Tehran. Data collection was done through two stages. The first stage was done before the height of the sanctions in July 2012 and we collected data from 40 community pharmacies. The second stage took place in March 2013, when the sanctions’ effects were quite evident on the general economy indicated by high inflation and reports of shortages in the media. We collected data again from all the pharmacies included in the first stage.

*Sampling*

A list of the all community pharmacies in 22 council districts of Tehran was prepared. Then, 110 pharmacies were selected randomly from this list. As a pilot assessment of the tool and methods, 40 pharmacies from this list were approached alphabetically to collect the data. After 1 year, in the second stage we realized that because of the sanction, the availability of the medicines may has been decreased. Therefore, to test the impact of sanctions on study outcomes (as secondary outcome of the main study), we repeated the study on 40 pharmacies randomly selected from those enrolled in the first step. 

*Data collection tool*

We first obtained the list of all the asthma medicines registered in the national formulary of Iran, including generic products, brand generic, imported brand generic and imported brand medicines. We developed a questionnaire containing Questions following the standard methodology proposed by the WHO and Health Action International (12). The preliminary questionnaires were assessed in terms of face validity and readability by pharmacists, a pulmonologist with experience of asthma questionnaires, and three health service researchers. The questionnaires were assessed in a pilot study and updated and shortened as a result.

*Data Collection and analysis*

All of the data were collected by one of the authors (GGh) who is a pharmacist. Data collection involved face-to-face interviews with pharmacists or medicine dispensers in each pharmacy and involved observing the pharmacy shelves for available medicines. 

Collected data were examined during the data collection period in order to ensure the accuracy of the data. To ensure data validity we checked some data again (via phone) randomly. We used the student’s t-test to analyze the data, while categorizing the medicines into the following groups: imported branded generic and, domestically produced generic or brand-generic medicines. We also assessed the availability of any inhaled corticosteroid medicines (whether brand or generic) or any combination of an inhaled corticosteroid and beta-agonist medicines (whether brand or generic). The latter was to assess whether the pharmacies had the minimum medicines required to provide the WHO list of essential medicines for asthma. salbutamol and Beclometason are in the essential list of WHO. 

## Results and Discussion

In total, there were 17 medicinal molecules used for asthma care registered in Iran that amounted to 10 generic or brand-generic products and 18 imported medicinal forms. As it is observed in Tables 1 and 2, the availability of imported and locally produced medicines in Tehran substantially declined after the height of the sanctions. The reduction in availability was starker for imported medicines, where the relative difference ranged from 67 to 86% reduction in availability (Table 1.). 

We observed a similar substantial reduction in the availability of locally manufactured asthma medicines. The relative difference in availability of medicines was reduced in a range from 20 to 62% (Table 2.).

In total, while before the height of the sanctions 80% of the observed pharmacies provided at least one inhaled corticosteroid medicine and 60% provided at least one combination of an inhaled corticosteroid and an inhaled beta-agonist, the corresponding figures reduced to 33 and 28 percent after the sanctions (p- values: <0.0001 and 0.003 respectively) (Table 3.).

## Conclusion

We investigated the availability of asthma medicines in 40 randomly chosen community pharmacies of Tehran before and after the economic sanctions. We observed a dramatic reduction in the availability of medicines in the community pharmacies. The proportion of pharmacies that had at least one inhaled beta-agonist and one corticosteroid inhaler (i.e. essential medicines for asthma) at the time of the second visit reduced from 60% to 28%. The impact expectedly affected the imported brand medicines, but was also noted for locally produced generics and brand generics. The latter group of medicines were affected probably because the local manufacturers relied upon imported raw materials, excipients and the packaging requirements (13, 14). We also observed that availability to both locally manufactured and imported medicines for asthma was already less than before the sanctions; however, the decline in the availability of pharmacies to both locally manufactured and imported asthma medicines was considerable after the sanctions. 

In most developed countries, the mortality rate regarding asthma has been decreased due to using appropriate medicines (15, 16). Thus, asthma treatments improve physical functioning, the quality of life, and morbidity to a large extent and if they are used properly costs of health care can be reduced significantly as well (17). However, the results of this work indicate that access to necessary medicines has reduced considerably in Iran after the international sanctions were made, probability resulting in an increase in patients’ suffering. Hence, these economic sanctions exacerbated public health conditions through limiting their access to basic needs such as medical supplies. Additionally, shrinkage in national economy, inflation and deterioration of the financial status of the households may reduce the affordability of even available medicines and health services (18)
